# Effects of Metabolic Syndrome on Cognitive Performance of Adults During Exercise

**DOI:** 10.3389/fpsyg.2019.01845

**Published:** 2019-08-08

**Authors:** Marco Guicciardi, Antonio Crisafulli, Azzurra Doneddu, Daniela Fadda, Romina Lecis

**Affiliations:** ^1^Department of Pedagogy, Psychology and Philosophy, Faculty of Humanities, University of Cagliari, Cagliari, Italy; ^2^Sports Physiology Laboratory, University of Cagliari, Cagliari, Italy

**Keywords:** metabolic syndrome, exercise, cognitive processes, NIRS, attentional task

## Abstract

The metabolic syndrome (MS) has been associated with poor performances in multiple cognitive domains, as processing speed, visuo-spatial abilities, and executive functioning. Exercise is a critical factor for MS people’s vulnerability to cognitive dysfunction, because this may be beneficial to reduce cognitive impairment, but limited physical activity and impaired cerebral blood flow in response to exercise have been reported by individuals suffering from MS. Using an attentional interference test, the Bivalent Shape Task (BST), and metaboreflex, we analyzed cognitive performance and cerebral oxygenation (COX) in 13 MS people (five women), and 14 normal age-matched control (CTL, six women). Five different sessions were administered to all participants, each lasting 12 min: control exercise recovery (CER), post-exercise muscle ischemia (PEMI) to activate the metaboreflex, CER + BST, PEMI + BST, and BST alone. During each session, cognitive performance was assessed by means of response times and response accuracy with which participants make the decision and COX was evaluated by near infrared spectroscopy with sensors applied in the forehead. Compared to CTL, MS group performed significantly worse in all sessions (*F* = 4.18; *p* = 0.05; ES = 0.13): their poorest performance was observed in the BST alone session. Moreover, when BST was added to PEMI, individuals of the CTL group significantly increased their COX compared to baseline (103.46 ± 3.14%), whereas this capacity was impaired in MS people (102.37 ± 2.46%). It was concluded that: (1) MS affects cognitive performance; (2) people with MS were able to enhance COX during exercise, but they impair their COX when an attentional interference task was added.

## Introduction

The metabolic syndrome (MS) is a cluster of interrelated conditions, increasing the risk of heart disease, heart disease, Type 2 diabetes mellitus, and stroke, among other health problems. The International Diabetes Federation estimated that 20–25% of the global adult population suffers from MS (International Diabetes Federation, 2015), which increases in older adults, but rates of MS ranging between 0 and 19.2% were also reported among children and adolescents (Friend et al., 2013). According to clinical guidelines, an individual suffers from MS if three or more risk factors as high blood glucose, high blood pressure, high plasma triglycerides, high waist circumference, and low HDL cholesterol are present (Expert Panel on Detection, Evaluation, and Treatment of High Blood Cholesterol in Adults, 2001; Alberti et al., 2009).

The MS has been correlated to cognitive dysfunction and poor performances in multiple domains as processing speed, memory, semantic fluency, visuo-spatial abilities, sustained attention, and executive function (Yaffe et al., 2004; Segura et al., 2009; van den Berg et al., 2009; Solfrizzi et al., 2011; Yates et al., 2012; Wooten et al., 2019). In order to explain the effects of MS on cognitive impairments different risk factors such as neuroinflammation, oxidative stress, impaired vascular reactivity, and abnormal brain lipid metabolism have been taken into account (Yates et al., 2012).

Although every single risk factor plays some role in the decline of cognitive function, the question was raised as to whether these risk factors taken together have a higher predictive value for cognitive deterioration. Cohort studies with cross-sectional research designs (Yaffe et al., 2004; Segura et al., 2009; Vieira et al., 2011) or longitudinal (Yaffe et al., 2009; Akbaraly et al., 2010) build a strong case for MS being associated with poor cognitive performance than its individual components. MS is mostly associated with impaired executive functioning, among all reported deficits (Wooten et al., 2019), although findings are mixed, with some cross-sectional studies reporting no effects (Tournoy et al., 2010; Kim et al., 2011) or even better cognitive performance among older women suffering from MS (Laudisio et al., 2008). Clearly, some of the ambiguity in the results obtained could be the effect of methodological issues as cognitive domains selected, demographic characteristics (i.e., differences in age, race, gender, educational level, and socioeconomic position), MS status vs. persistent MS, health status of experimental and control group, and complexity in splitting the impact of individual factors from that of the MS itself (Akbaraly et al., 2010); further studies incorporating some protective factors have been also claimed (Yates et al., 2012).

The vascular risk factors as, for example, high blood sugar and low level of HDL cholesterol, are all potentially reversible and current recommendations for the treatment of MS encourage intensive therapeutic lifestyle changes such as increasing regular physical activity and reducing the dietary intake of saturated fat and cholesterol. Regular physical activity of moderate intensity can reduce singular metabolic risk factors while benefits of exercise on multiple risk factors were also reported (Laaksonen et al., 2002; Katzmarzyk et al., 2003; Holme et al., 2007). Previous researches show evidence that a lower prevalence of MS is associated with at least 150 min weekly of moderate-intensity PA (Strasser, 2013). However, to appreciate the benefits of movement it is relevant to distinguish among physical activity and exercise: while physical activity refers to any movement produced by skeletal muscles that require energy, exercise enhances the capacity and efficiency of the cardiorespiratory system and muscular strength associated with health and functional capacity (Caspersen et al., 1985). Improvements in fitness are noticeable in enhancement in executive-control processes such as, inhibition, planning, scheduling, coordination, and working memory. Due to brain activation and to the increase of cerebral blood flow (CBF) exercise may be therefore beneficial to reduce cognitive impairment (Secher et al., 2008; Kim et al., 2011). Interventions, such as exercise training programs, increase the cognitive performance of one-half standard deviation on average, regardless of the type of training method, cognitive task, or participants’ characteristics (Colcombe and Kramer, 2003). Other potential mechanisms that account for the cognition-enhancing effects of exercise encompassed vascularization, neuroendocrine response to stress, neuroinflammation, brain amyloid burden, or other favorable effects on neuronal survivability and function (Baker et al., 2010).

Moreover, muscle metaboreflex activated by metabolites accumulating in the muscle during contraction can enhance the sympathetic tone and consequently the sympathetic nervous system (SNS) activity (Boushel, 2010). However, in people with metabolic disorders impaired CBF was reported whether caused by exercise (Kim et al., 2015; Vianna et al., 2015) or by stimulation of metaboreflex (Delaney et al., 2010; Milia et al., 2015; Crisafulli, 2017). Consequently, we have assumed that exercise is a critical factor for MS people’s vulnerability to cognitive dysfunction, because may support cognitive functions, but limited physical activity and impaired CBF after exercise has been reported by individuals suffering from MS. When cerebral oxygenation (COX) is reduced after exercise (or stimulation of metaboreflex) cognitive functions can deteriorate and people experience early fatigue (González-Alonso et al., 2004; Rasmussen et al., 2010). Subclinical alterations in cerebrovascular reactivity and cerebral metabolism may denote early brain compromise associated with peripheral metabolic disturbances (Yates et al., 2012). Individuals with MS may not be able to retain an optimal neuronal environment, mostly when a high demanding cognitive task is superimposed to exercise. We hypothesized therefore that the association between metaboreflex and a contemporary mental task could result in an impairment of cognitive performance and in a reduced COX, in people suffering from MS.

## Materials and Methods

### Participants

Two groups of participants were enrolled:

• MS group: 13 patients [five women, mean ± standard deviation (SD) of the mean of age 52.9 ± 11.2 years], who received a diagnosis of MS from at least a year (range 1–6 years). To be included in the study individual have to present three or more of the following five metabolic factors: (1) waist circumference >102 and >88 cm for men and women, respectively; (2) high blood pressure (≥130/85 mmHg); (3) low HDL cholesterol (≤40 and ≤50 mg/dL in men and women, respectively); (4) high triglycerides (≥150 mg/dL); and (5) high fasting glucose level (≥100 mg/dL).
• Control (CTL) group: 14 age-matched healthy participants (six women, mean ± SD of age 50.8 ± 8.1 years), unaffected by any metabolic disease as resulted from anamnesis and physical examination.

In addition to age ≤18 and ≥65 years, exclusion criteria encompassed significant medical disease that could interfere with the autonomic function. Smokers and individuals taking sympatho-mimetics, β-blockers, and/or tricyclic antidepressants were also excluded.

### Procedure

After enrolment, participants attended the laboratory on two occasions: one preliminary baseline visit and one experimental phase.

Baseline visit: All participants received a medical examination with anamnesis, anthropometric measures (weight, height, body mass index, waist circumference, and body composition), ECG, and blood pressure. To assess physical capacity all participants performed a cardiopulmonary exercise test (CPT) on a cycle ergometer (CUSTO Med, Ottobrunn, Germany): maximum heart rate (HRmax), maximal oxygen uptake (VO2max), and maximum workload (Wmax) were recorded. During the baseline visit, the participants familiarized with the laboratory equipment and staff.

**FIGURE 1:**
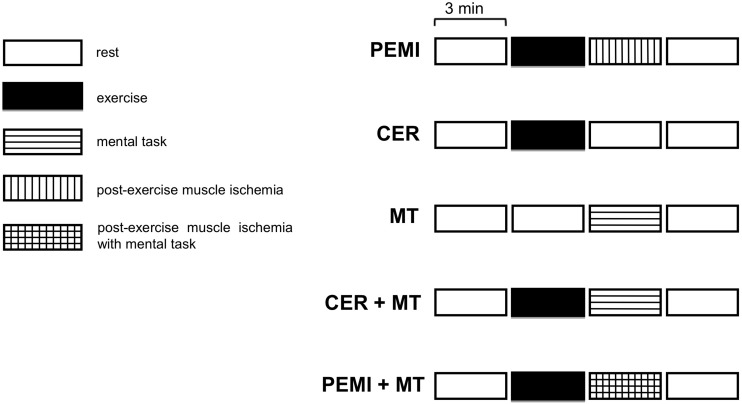
Schematic representation of the various sessions of the study protocol. PEMI, post-exercise muscle ischemia; CER, control exercise recovery; MT, mental task; CER + MT, control exercise recovery + mental task; PEMI + MT, post-exercise muscle ischemia + mental task.

Experimental phase: this phase started after at least 3 days (range 3–7 days) from baseline visit and included in random order five sessions (Figure 1). All participants were randomly assigned to the following five sessions. All sessions were composed of four blocks each lasting 3 min, for a total of 12 min. Sessions were spaced by at least 15 min of recovery. Recovery was considered complete when the heart rate was not higher than 5 bpm compared with the pre-exercise level. Participants were requested to abstain from alcohol, caffeinated beverages, and heavy exercise for 12 h prior to coming to the laboratory.

This study was carried out in accordance with the recommendations of the Code of Ethics for Research in Psychology, Italian Association of Psychology. The protocol was approved by the ethics committee of the University of Cagliari. All participants gave written informed consent in accordance with the Declaration of Helsinki.

The experimental phase started after at least 3 days (range 3–7 days) from baseline visit and included in random order the following five sessions (Figure 1):

Specifically, sessions were as follows:

• Post-exercise muscle ischemia (PEMI): 3 min of resting, followed by 3 min of exercise, consisting of rhythmic (30 compressions/min). The handgrip was settled at 30% of the maximum previously assessed as the peak reached during five maximal compressions executed on a hydraulic dynamometer (MAP 1.1, Kern, Balingen, Germany). The exercise was followed by 3 min of PEMI on the exercised arm induced by rapidly inflating an upper arm biceps tourniquet to 50 mmHg above peak exercise systolic pressure. The cuff was kept inflated for 3 min. Three minutes of recovery was further allowed after the cuff was deflated, for a total of 6 min of recovery.
• Control exercise recovery (CER): the same rest-exercise protocol used for PEMI was performed followed by a CER of 6 min without tourniquet inflation. The CER session was employed to have a control recovery situation without metaboreflex activation.
• Mental task (MT): A computerized attentional interference test, the Bivalent Shape Task (BST) (Esposito et al., 2013), was used to test the ability to suppress interference. The BST asks the participant to decide whether a shape at the center of the screen is a square or a circle. The stimulus shape is presented either in blue, red, or an unfilled black outline. Visual response cues are provided below the stimulus, indicating the side of the response and are shaded in either red or blue. Three trial types were presented: neutral trials in which the stimulus is black and white; congruent trials, in which the (irrelevant) color of the stimulus matches the response cue; and incongruent, in which the (irrelevant) color mismatches the response cue. Compared to the condition in which the dominant response and the activated response are the same (e.g., congruent trials), a response interference is stimulated whenever the dominant response has to be suppressed in order to give the instructed response, as in the incongruent trials: this results in a deterioration of the performances. Dependent measures are the speed and accuracy with which participants take the decision: the speed was assessed in ms, the accuracy as percent of correct responses. The MT started after 6 min of rest and lasted for 3 min. Three further minutes of recovery was allowed after the termination of MT.
• CER + MT: the same rest-exercise protocol used for CER was performed. The exercise phase was followed by a MT session of 3 min, i.e., the same duration of the MT session described previously. Three further minutes of recovery was allowed after the termination of MT.
• PEMI + MT: the same rest-exercise protocol used for PEMI was performed. The exercise phase was followed by 3 min of contemporary PEMI and MT. Three further minutes of recovery was allowed after the termination of the PEMI + MT period.

Throughout sessions, COX was assessed by means of near infrared spectroscopy (NIRS; Nonin, SenSmart X-100, Plymouth, MN, United States), which provided a measure of oxygenated Hb in the brain tissue. It is recognized that changes in COX are representative of cortical activation (Strangman et al., 2002) and COX assessments by means of NIRS during mental tasks such as calculation, interference, or Stroop tasks were already reported (Plichta et al., 2006; Verner et al., 2013; Ferreri et al., 2014). Two NIRS sensors were placed on the right and left subject’s forehead above the eyebrow, in the regions between Fp1 and F3 (international EEG 10–20 system) and adjusted according to the strong signal. Oxygenated Hb measured by NIRS can be considered an index of regional tissue blood flow as previously reported (Suzuki et al., 2004). Since the absolute concentration of oxygenated Hb could not be acquired, because the path length of NIRS light within the brain tissue was unknown, relative changes of NIRS signals against the baseline values were taken into account.

This study was carried out in accordance with the recommendations of the Code of Ethics for Research in Psychology, Italian Association of Psychology. The protocol was approved by the ethics committee of the University of Cagliari. All participants gave written informed consent in accordance with the Declaration of Helsinki.

### Data Analysis

Preliminary analysis showed that all the BST protocols were valid with an accuracy of responses equal or above the predetermined threshold. A preliminary check of data, executed by means of the Kolmogorov–Smirnov test with Lilliefors correction that renders this test less conservative, has confirmed a normal distribution for all parameters examined. The *t*-test for independent groups was used to assess differences in anthropometric characteristics, levels of triglycerides, HDL cholesterol, fasting glucose, and results of the CPT. Two-way ANOVA (factors: group and condition) was executed to assess (a) response times (ms) on BST; (b) change in COX reported as % changes from rest. Statistics were carried out utilizing commercially available software (GraphPad Prism and SPSS ver. 17.0). Statistical significance was established as a *p*-value of <0.05 in all cases.

## Results

The protocol was completed by all participants none reported discomfort or unbearable pain during PEMI. Anthropometric characteristics of both groups together with results of the baseline visit are shown in Table 1.

**TABLE 1:** Anthropometric characteristics of both groups together with results of the screening medical examination and of cardiopulmonary test.

**Anthropometric characteristics**	**CTL**	**MS**	***P*-value**
Height (cm)	169.5 ± 10.29	165.92 ± 8.08	0.326
Body mass (kg)	69.45 ± 12.23	96.73 ± 14.13	<0.001
Body mass index (kg/m^2^)	24.03 ± 2.74	35.26 ± 5.65	<0.001
Waist circumference (cm)	80.75 ± 9.85	113.58 ± 7.24	<0.001
Fat mass (%)	21.8 ± 5.5	35.9 ± 7.3	<0.001
Fat-free mass (%)	78.2 ± 5.5	64.1 ± 7.3	<0.001
Total body water (%)	57.1 ± 4.5	47.3 ± 5.5	<0.001
Systolic blood pressure (mmHg)	111.07 ± 9.84	123.85 ± 8.69	0.001
Diastolic blood pressure (mmHg)	73.57 ± 6.91	81.15 ± 6.50	0.007
Maximal O_2_ uptake (mL/kg/min)	31.70 ± 9.36	19.99 ± 3.46	<0.001
Maximum workload (W)	199.30 ± 84.39	140.76 ± 29.56	0.026
Maximum heart rate (bpm)	158.80 ± 12.45	147.84 ± 13.15	0.035

*Values are mean ± SD. CTL = controls (*n* = 14), MS = Metabolic Syndrome patients (*n* = 13).*

Participants of the MS group had a higher weight, BMI, waist circumference, FM, SBP, DBP, and fasting glucose with respect to the CTL group. Table 1 also shows that FFM, TBW, VO2max, Wmax, and HRmax were lower in the MS than in the CTL group.

Table 2 shows that the significant differences between groups were found only in BST scores, while COX was significantly affected by the condition. In particular, MS group, compared to CTL group, performed significantly worse in all sessions were MT was included (*F* = 4.18; *p* = 0.05; ES = 0.13). In both groups, COX increased during the CER, the PEMI, the CER + MT, and the PEMI + MT tests compared to the MT test.

**TABLE 2:** Bivalent shape test (BST) scores and cerebral oxygenation (COX) during the mental task (MT), control exercise recovery + mental task (CER + MT), and post-exercise muscle ischemia + mental task (PEMI + MT) tests for control (CTL, *n* = 14) and metabolic syndrome (MS, *n* = 13) groups.

**Anthropometric characteristics**	**MT**	**CER + MT**	**PEMI + MT**	***P-*value condition effect**	***P-*value group effect**	***P-*value interaction**
BST (ms)	CTL1020.05 ± 271.74	CTL970.99 ± 271.74	CTL987.15 ± 224.54	0.311	0.050	0.857
	MS1249.00 ± 444.35	MS1235.45 ± 380.69	MS1201.96 ± 406.34			
COX (%)	CTL100.79 ± 1.41	CTL102.61 ± 1.56^∗^	CTL103.3 ± 2.09^∗^	<0.001	0.969	0.524
	MS100.36 ± 1.10	MS103.10 ± 2.89^∗^	MS102.54 ± 1.27^∗^			

*Values are mean ± SD. ^∗^*p* < 0.05 vs. MT test of the same group.*

**FIGURE 2:**
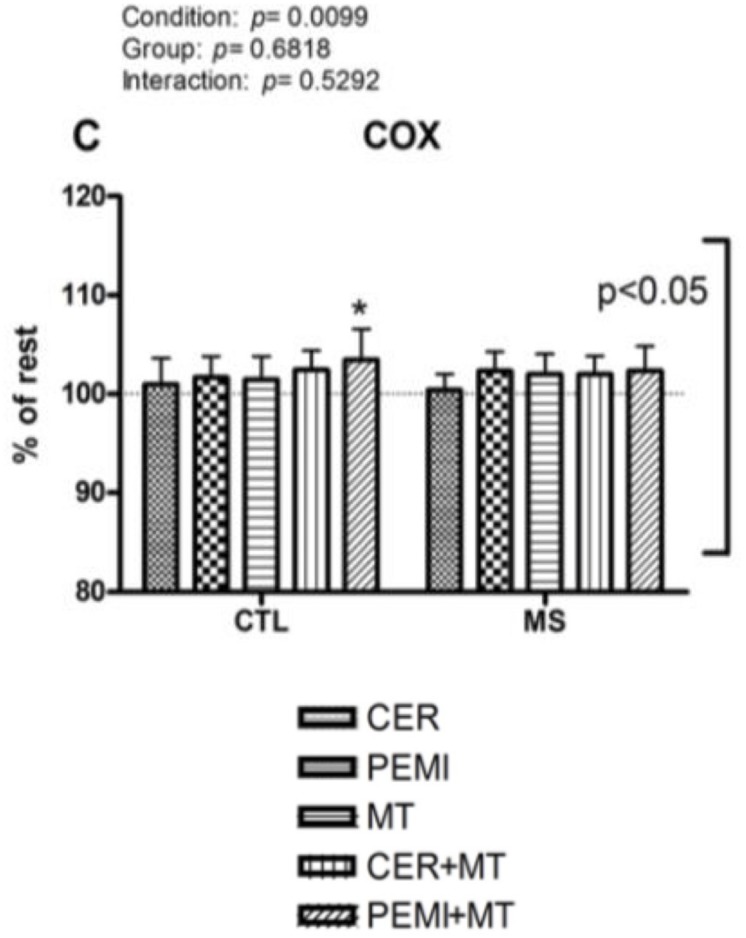
Cerebral oxygenation data during control exercise recovery (CER), post-exercise muscle ischemia (PEMI), mental task (MT), control exercise recovery + mental task (CER + MT), and post-exercise muscle ischemia + mental task (PEMI + MT) tests in the metabolic syndrome (MS, *n* = 13) and the control (CTL, *n* = 14) groups. The dotted line indicates rest level. Values are mean ± SD. A horizontal bracket indicates the overall main effect of groups; a vertical bracket indicates the overall main effect of condition. ^∗^*p* < 0.05 vs. CER test of the same group.

Moreover, when MT was added to PEMI, individuals of the CTL group significantly increased their COX compared to baseline (103.46 ± 3.14%), whereas this capacity was impaired in MS people (102.37 ± 2.46%, see Figure 2).

## Discussion

The management of chronic diseases requires to assume and maintain an active role centered on: (a) acquisition of self-regulated behavior; (b) suppression of interferent habitual response (such as sitting after meals); and (c) replacement of habit with other healthier behavior (such as going for a walk) (Settineri et al., 2019). Exercise appears to be a useful strategy to treat MS, because it could reduce cognitive impairment and risk to develop Type 2 diabetes mellitus, stroke, and heart diseases. However, adults with MS may be at increased risk of non-adherence exercise due to a concurring rise in behavioral requests (e.g., nutrition planning, glucose tracking, medication timing) and decrease in cognitive function (Olson et al., 2017). Compared to modifications of nutrition or taking medications, exercise requires more time to be practice and deliberate continuous effort of planning and monitoring: people suffering from chronic diseases often perceive its adoption as a significant and difficult change in their lifestyle (Guicciardi et al., 2014).

The principal aim of the present investigation was to characterize the mental performance and the COX in people suffering from MS during exercise, associating a mental task (BST) to metaboreflex activated by means of the PEMI method.

About the mental performance, significant differences in response times were found between groups: the participants suffering from the MS took longer to discriminate the shapes of BST. Compared to CTL group, their performances were always worse, supporting the hypothesis that MS can impair executive functions, such as interference suppression. The difference in cognitive performance due to MS was small (ES = 0.13); however, considering the high prevalence of MS, the burden of impaired cognition due to the MS may be substantial in the overall population (Vieira et al., 2011). The individuals suffering from MS may not be able to maintain an optimal performance during high demanding tasks as BST, which requires participants to control the interference due to the stimulus characteristic (e.g., color) and focus on the relevant information (e.g., shape) to give the correct response. Both groups have reported their poorest cognitive performance in the MT alone trial, but when PEMI or CER was superimposed their response times improved even if not significantly. As know, exercise has more effect on executive functions, than on any other type of cognitive process (Colcombe and Kramer, 2003); however, to evaluate concurrent improvements in other cognitive processes, such as working memory, sustained attention, or visuo-spatial abilities further researches should be conducted using more suitable tasks.

About the COX, participants suffering from MS, compared with healthy controls, were unable to increase COX when BST was superimposed to the PEMI-induced metaboreflex activation. This occurrence suggests that people suffering from MS were not able to increase COX to the same extent of CTL groups when a mental task was superimposed to the PEMI-induced metaboreflex activation. In both groups during the mental task, COX tended to slightly increase with respect to the baseline. This MT-induced raise in COX was probably the consequence of the increased brain activity due to the mental task, which enhanced brain metabolism and led to COX elevation (Willie et al., 2014). All COX levels were similar among conditions with an exception: during the PEMI + MT test of the CTL group, COX was significantly more elevated than the baseline. This could demonstrate that adding a mental task to the PEMI-induced metaboreflex activation increased COX in normal individuals, but not in individuals suffering from MS, who were not able to increase COX to the same extent of CTL participants.

We examined the effects of experimental sessions on response times and we used these data to infer information about the brain mechanisms involved while performing a mental task during an exercise. We cannot provide any ultimate explanation for the lacking increase of COX in MS group when a mental task was superimposed to PEMI: individuals could have an elevated SNS drive which impaired the capacity to enhance COX or an impaired cerebral vascular reactivity or a reduced capacity to maintain COX at a proper level. Whatever the cause results suggest that individuals suffering from MS have impaired executive control and are unable to properly increase COX when a mental task activity was superimposed the metaboreflex obtained by means of PEMI maneuver. This phenomenon could explain why individuals suffering from MS do not meet physical activity recommendation, since executive functions are necessary for behavior change and can affect an individual’s capability to successfully adopt and maintain physical activity behavior change (Olson et al., 2017).

### Limitations of the Study

One possible limitation of this study is the response times of BST assumed as proxies to assess executive functions. Attention and processing speed are typically categorized as a general cognitive function; however, they are more related to executive function domain and to a successful behavioral change (Olson et al., 2017). The BST is an attentional test, where color and shape are varied within trials. The task is similar to other traditional interference tasks (e.g., the Simon interference task, the color-word Stroop test, and Eriksen’s flanker task), but not prompts verbal reactions. The task was already used to assess the ability to suppress irrelevant information in non-linguistic settings, but more studies are required to validate this task with adults suffering from MS. Another possible limitation of the present investigation is that SNS activity was not directly assessed. This kind of measure are somewhat invasive and not applicable in experimental settings such as the present one, with ongoing psychological stress. A further potential limit of this study is related to the use of NIRS, compared to other brain imaging techniques, such as transcranial Doppler (TCD) or functional magnetic resonance imaging (FMRI). Several studies have confirmed the sensitivity of NIRS to measure subtle changes in COX during arithmetic or neuropsychological tasks involving the frontal lobe such as Stroop test (Ferreri et al., 2014). However, NIRS suffers of low spatial resolution which makes difficult to detect changes in areas located distant from the monitored site. On the other hand, NIRS offers some advantages as portability, good motion tolerance, non-invasiveness, and low maintenance costs. A further limitation of this study is the small size of the sample of individuals suffering from MS, that asks for studies replications. Finally, we cannot exclude that asking participants to refrain from caffeinated beverages, alcohol, and heavy exercise for 12 h before the experimental phase, may have affected their routine, and therefore every participant’s average performance.

## Conclusion

In conclusion, the results of the present investigation provide evidence that individuals suffering from MS had worse cognitive performance, compared to control group, in an attentional interference task, which involves executive functions. Individuals with MS were able to enhance COX during handgrip and during the metaboreflex activation; however, they could not further enhance COX when a mental task was superimposed to the metaboreflex. This conclusion could be intriguing as it may provide a potential psycho–physio–pathological basis of the scarce predisposition to exercise often reported in people with MS, since metaboreflex and cognitive performance are both operating during exercise (Wasmund et al., 2002) and executive functions are necessary to initiate and maintain physical activity behavior change (Olson et al., 2017).

## Data Availability

The datasets generated for this study are available on request to the corresponding author.

## Author Contributions

The final version of this manuscript was written by MG and AC who contributed equally to the theoretical and empirical aspects of the study. AD collected and analyzed the medical data and wrote a preliminary version of this manuscript. RL and DF, respectively, collected and analyzed the psychological data.

## Conflict of Interest Statement

The authors declare that the research was conducted in the absence of any commercial or financial relationships that could be construed as a potential conflict of interest.

## References

[B1] Akbaraly T. N. Kivimaki M. Shipley M. J. Tabak A. G. Jokela M. Virtanen M. (2010). Metabolic syndrome over 10 years and cognitive functioning in late midlife: the Whitehall II study. *Diabetes Care* 33 84–89. 10.2337/dc09-1218 19837794 PMC2797991

[B2] Alberti K. G. Eckel R. H. Grundy S. M. Zimmet P. Z. Cleeman J. I. Donato K. A. (2009). Harmonizing the metabolic syndrome: a joint interim statement of the international diabetes federation task force on epidemiology and prevention; national heart, lung, and blood institute; american heart association; world heart federation; international atherosclerosis society; and international association for the study of obesity. *Circulation* 120 1640–1645. 10.1161/CIRCULATIONAHA.109.192644 19805654

[B3] Baker L. D. Frank L. L. Foster-Schubert K. Green P. S. Wilkinson C. W. McTiernan A. (2010). Effects of aerobic exercise on mild cognitive impairment: a controlled trial. *Arch. Neurol.* 67 71–79. 10.1001/archneurol.2009.307 20065132 PMC3056436

[B4] Boushel R. (2010). Muscle metaboreflex control of the circulation during exercise. *Acta Physiol.* 199 367–383. 10.1111/j.1748-1716.2010.02133.x 20353495

[B5] Caspersen C. J. Powell K. E. Christenson G. M. (1985). Physical activity, exercise, and physical fitness: definitions and distinctions for health-related research. *Public Health Rep.* 100 126–131. 3920711 PMC1424733

[B6] Colcombe S. Kramer A. F. (2003). Fitness effects on the cognitive function of older adults: a meta-analytic study. *Psychol. Sci.* 14 125–130. 10.1111/1467-9280.t01-1-01430 12661673

[B7] Crisafulli A. (2017). The impact of cardiovascular diseases on cardiovascular regulation during exercise in humans: studies on metaboreflex activation elicited by the post-exercise muscle Ischemia method. *Curr. Cardiol. Rev.* 13 293–300. 10.2174/1573403X13666170804165928 28782491 PMC5730962

[B8] Delaney E. P. Greaney J. L. Edwards D. G. Rose W. C. Fadel P. J. Farquhar W. B. (2010). Exaggerated sympathetic and pressor responses to handgrip exercise in older hypertensive humans: role of the muscle metaboreflex. *Am. J. Physiol. Heart Circ. Physiol.* 299 H1318–H1327. 10.1152/ajpheart.00556.2010 20802135 PMC2993192

[B9] Esposito A. G. Baker-Ward L. Mueller S. (2013). Interference suppression vs. response inhibition: an explanation for the absence of a bilingual advantage in preschoolers’ stroop task performance. *Cogn. Dev.* 28 354–363. 10.1016/j.cogdev.2013.09.002 24453405 PMC3894626

[B10] Expert Panel on Detection, Evaluation, and Treatment of High Blood Cholesterol in Adults (2001). Executive summary of the third report of the national cholesterol education program (NCEP) expert panel on detection, evaluation, and treatment of high blood cholesterol in adults (Adult Treatment Panel III). *JAMA* 285 2486–2497. 10.1001/jama.285.19.2486 11368702

[B11] Ferreri L. Bigand E. Perrey S. Bugaïska A. (2014). The promise of near-infrared spectroscopy (NIRS) for psychological research: a brief review. *LAnnee Psychol.* 114 537–569. 10.4074/s0003503314003054

[B12] Friend A. Craig L. Turner S. (2013). The prevalence of metabolic syndrome in children: a systematic review of the literature. *Metab. Syndr. Relat. Disord.* 11 71–80. 10.1089/met.2012.0122 23249214

[B13] González-Alonso J. Dalsgaard M. K. Osada T. Volianitis S. Dawson E. A. Yoshiga C. C. (2004). Brain and central haemodynamics and oxygenation during maximal exercise in humans. *J. Physiol.* 557(Pt 1), 331–342. 10.1113/jphysiol.2004.060574 15004212 PMC1665053

[B14] Guicciardi M. Lecis R. Anziani C. Corgiolu L. Porru A. Pusceddu M. (2014). Type 2 diabetes: negative thoughts to physical activity. *Sport Sci. Health* 10 247–251. 10.1007/s11332-014-0201-1 PMC4346010 25750816

[B15] Holme I. Tonstad S. Sogaard A. J. Larsen P. G. L. Haheim L. L. (2007). Leisure time physical activity in middle age predicts the metabolic syndrome in old age: results of a 28-year follow-up of men in the Oslo study. *BMC Public Health* 7:154. 10.1186/1471-2458-7-154 17625024 PMC1947967

[B16] International Diabetes Federation (2015). *Diabetes Atlas 7th Edition.* Brussels, Belgium: International Diabetes Federation.

[B17] Katzmarzyk P. T. Leon A. S. Wilmore J. H. Skinner J. S. Rao D. C. Rankinen T. (2003). Targeting the metabolic syndrome with exercise: evidence from the HERITAGE family study. *Med. Sci. Sports Exerc.* 35 1703–1709. 10.1249/01.MSS.0000089337.73244.9B 14523308

[B18] Kim S.-H. Kim M. Ahn Y.-B. Lim H.-K. Kang S.-G. Cho J.-H. (2011). Effect of dance exercise on cognitive function in elderly patients with metabolic syndrome: a pilot study. *J. Sports Sci. Med.* 10 671–678. 24149557 PMC3761497

[B19] Kim Y.-S. Seifert T. Brassard P. Rasmussen P. Vaag A. Nielsen H. B. (2015). Impaired cerebral blood flow and oxygenation during exercise in type 2 diabetic patients. *Physiol. Rep.* 3:e12430. 10.14814/phy2.12430 26109188 PMC4510631

[B20] Laaksonen D. E. Lakka H.-M. Salonen J. T. Niskanen L. K. Rauramaa R. Lakka T. A. (2002). Low levels of leisure-time physical activity and cardiorespiratory fitness predict development of the metabolic syndrome. *Diabetes Care* 25 1612–1618. 10.2337/diacare.25.9.1612 12196436

[B21] Laudisio A. Marzetti E. Pagano F. Cocchi A. Franceschi C. Bernabei R. (2008). Association of metabolic syndrome with cognitive function: the role of sex and age. *Clin. Nutr.* 27 747–754. 10.1016/j.clnu.2008.07.001 18715681

[B22] Milia R. Velluzzi F. Roberto S. Palazzolo G. Sanna I. Sainas G. (2015). Differences in hemodynamic response to metaboreflex activation between obese patients with metabolic syndrome and healthy subjects with obese phenotype. *Am. J. Physiol. Heart Circ. Physiol.* 309 H779–H789. 10.1152/ajpheart.00250.2015 26163444

[B23] Olson E. A. Mullen S. P. Raine L. B. Kramer A. F. Hillman C. H. McAuley E. (2017). Integrated social- and neurocognitive model of physical activity behavior in older adults with metabolic disease. *Ann. Behav. Med.* 51 272–281. 10.1007/s12160-016-9850-4 27844326 PMC5475366

[B24] Plichta M. M. Herrmann M. J. Ehlis A.-C. Baehne C. G. Richter M. M. Fallgatter A. J. (2006). Event-related visual versus blocked motor task: detection of specific cortical activation patterns with functional near-infrared spectroscopy. *Neuropsychobiology* 53 77–82. 10.1159/000091723 16511338

[B25] Rasmussen P. Nielsen J. Overgaard M. Krogh-Madsen R. Gjedde A. Secher N. H. (2010). Reduced muscle activation during exercise related to brain oxygenation and metabolism in humans. *J. Physiol.* 588(Pt 11), 1985–1995. 10.1113/jphysiol.2009.186767 20403976 PMC2901984

[B26] Secher N. H. Seifert T. Van Lieshout J. J. (2008). Cerebral blood flow and metabolism during exercise: implications for fatigue. *J. Appl. Physiol.* 104 306–314. 10.1152/japplphysiol.00853.2007 17962575

[B27] Segura B. Jurado M. A. Freixenet N. Albuin C. Muniesa J. Junqué C. (2009). Mental slowness and executive dysfunctions in patients with metabolic syndrome. *Neurosci. Lett.* 462 49–53. 10.1016/j.neulet.2009.06.071 19560512

[B28] Settineri S. Frisone F. Merlo E. M. Geraci D. Martino G. (2019). Compliance, adherence, concordance, empowerment, and self-management: five words to manifest a relational maladjustment in diabetes. *J. Multidiscipl. Healthcare* 12 299–314. 10.2147/JMDH.S193752 31118655 PMC6499139

[B29] Solfrizzi V. Scafato E. Capurso C. D’Introno A. Colacicco A. M. Frisardi V. (2011). Metabolic syndrome, mild cognitive impairment, and progression to dementia. The Italian Longitudinal Study on Aging. *Neurobiol. Aging* 32 1932–1941. 10.1016/j.neurobiolaging.2009.12.012 20045217

[B30] Strangman G. Boas D. A. Sutton J. P. (2002). Non-invasive neuroimaging using near-infrared light. *Biol. Psychiatry* 52 679–693. 10.1016/s0006-3223(02)01550-0 12372658

[B31] Strasser B. (2013). Physical activity in obesity and metabolic syndrome. *Ann. N. Y. Acad. Sci.* 1281 141–159. 10.1111/j.1749-6632.2012.06785.x 23167451 PMC3715111

[B32] Suzuki M. Miyai I. Ono T. Oda I. Konishi I. Kochiyama T. (2004). Prefrontal and premotor cortices are involved in adapting walking and running speed on the treadmill: an optical imaging study. *NeuroImage* 23 1020–1026. 10.1016/j.neuroimage.2004.07.002 15528102

[B33] Tournoy J. Lee D. M. Pendleton N. O’Neill T. W. O’Connor D. B. Bartfai G. (2010). Association of cognitive performance with the metabolic syndrome and with glycaemia in middle-aged and older European men: the European male ageing study. *Diabetes/Metab. Res. Rev.* 26 668–676. 10.1002/dmrr.1144 21043047

[B34] van den Berg E. Kloppenborg R. P. Kessels R. P. C. Kappelle L. J. Biessels G. J. (2009). Type 2 diabetes mellitus, hypertension, dyslipidemia and obesity: a systematic comparison of their impact on cognition. *Biochim. Biophys. Acta* 1792 470–481. 10.1016/j.bbadis.2008.09.004 18848880

[B35] Verner M. Herrmann M. J. Troche S. J. Roebers C. M. Rammsayer T. H. (2013). Cortical oxygen consumption in mental arithmetic as a function of task difficulty: a near-infrared spectroscopy approach. *Front. Hum. Neurosci.* 7:217. 10.3389/fnhum.2013.00217 23734120 PMC3660659

[B36] Vianna L. C. Deo S. H. Jensen A. K. Holwerda S. W. Zimmerman M. C. Fadel P. J. (2015). Impaired dynamic cerebral autoregulation at rest and during isometric exercise in type 2 diabetes patients. *Am. J. Physiol. Heart Circ. Physiol.* 308 H681–H687. 10.1152/ajpheart.00343.2014 25599569 PMC4385994

[B37] Vieira J. R. Elkind M. S. V. Moon Y. P. Rundek T. Boden-Albala B. Paik M. C. (2011). The metabolic syndrome and cognitive performance: the Northern Manhattan Study. *Neuroepidemiology* 37 153–159. 10.1159/000332208 22005335 PMC3214939

[B38] Wasmund W. L. Westerholm E. C. Watenpaugh D. E. Wasmund S. L. Smith M. L. (2002). Interactive effects of mental and physical stress on cardiovascular control. *J. Appl. Physiol.* 92 1828–1834. 10.1152/japplphysiol.00019.2001 11960930

[B39] Willie C. K. Tzeng Y. C. Fisher J. A. Ainslie P. N. (2014). Integrative regulation of human brain blood flow. *J. Physiol.* 592, 841–859. 10.1113/jphysiol.2013.268953 24396059 PMC3948549

[B40] Wooten T. Ferland T. Poole V. Milberg W. McGlinchey R. DeGutis J. (2019). Metabolic risk in older adults is associated with impaired sustained attention. *Neuropsychology* 10.1037/neu0000554 [Epub ahead of print]. 31094549

[B41] Yaffe K. Kanaya A. Lindquist K. Simonsick E. M. Harris T. Shorr R. I. (2004). The metabolic syndrome, inflammation, and risk of cognitive decline. *JAMA* 292 2237–2242. 10.1001/jama.292.18.2237 15536110

[B42] Yaffe K. Weston A. L. Blackwell T. Krueger K. A. (2009). The metabolic syndrome and development of cognitive impairment among older women. *Arch. Neurol.* 66 324–328. 10.1001/archneurol.2008.566 19273750 PMC2685462

[B43] Yates K. F. Sweat V. Yau P. L. Turchiano M. M. Convit A. (2012). Impact of metabolic syndrome on cognition and brain: a selected review of the literature. *Arterioscler. Thromb. Vasc. Biol.* 32 2060–2067. 10.1161/ATVBAHA.112.252759 22895667 PMC3442257

